# Perinatal Exposure to Heavy Metals and Trace Elements of Preterm Neonates in the NICU: A Toxicological Study Using Multiple Biomatrices

**DOI:** 10.3390/toxics13100898

**Published:** 2025-10-20

**Authors:** Melda Celik, Irem Iyigun, Siddika Songül Yalcin, Murat Cagan, Deniz Arca Cakir, Hasan Tolga Celik, Ozgur Deren, Pinar Erkekoglu

**Affiliations:** 1 Division of Social Pediatrics, Department of Pediatrics, Sıhhiye, 06100 Ankara, Türkiye; 2 Division of Neonatology, Department of Pediatrics, Hacettepe University Faculty of Medicine, Sıhhiye, 06100 Ankara, Türkiye; 3 Division of Perinatology, Department of Gynaecology and Obstetrics, Hacettepe University Faculty of Medicine, Sıhhiye, 06100 Ankara, Türkiye; 4 Department of Pharmaceutical Toxicology, Hacettepe University Faculty of Pharmacy, Sıhhiye, 06100 Ankara, Türkiye

**Keywords:** heavy metals, newborns, NICU, toxic, trace elements

## Abstract

In this study we aimed to investigate the levels of selected heavy metals and trace elements (Hg, Pb, Cd, As, Mn, Se, and Cu) in three different biomatrices—maternal urine (Mu), neonatal urine (Nu), and cord blood—of preterm newborns born at less than 35 weeks’ gestation who were staying in the NICU and their mothers, and the relationships of these elements with maternal and neonatal characteristics. Cord Pb, As, and Hg were significantly lower than in Mu, whereas Se and Cu were higher (*p* < 0.001). All elements were excreted more in Mu than in Nu (*p* < 0.001). Nu levels of Cd, Mn, Se, and Cu were lower, while As and Hg were higher than in cord blood. Nu metal excretion increased significantly over time (*p* < 0.001). Positive correlations were found between MuCu and NuCu (r_s_ = 0.35) and between maternal Se and maternal age (r_s_ = 0.41). NuHg, MuMn, and cord Mn showed negative correlations with penile length, and NuHg was also negatively correlated with anogenital distance. The first and second NuPb levels were positively correlated with birth weight percentile. The findings suggest transplacental transmission and ongoing exposure to heavy metals and trace elements in preterm infants, highlighting the importance of prenatal environmental exposure awareness for healthcare providers.

## 1. Introduction

Humans are widely exposed to environmental inorganic pollutants. Of these, heavy metals such as lead (Pb), cadmium (Cd), mercury (Hg), and arsenic (As) are among the most toxic substances, as ranked by the Agency for Toxic Substances and Disease Registry (ATSDR) of the Centers for Disease Control (CDC) [1]. The most common ways of human exposure are through drinking water and food, inhalation of cigarette smoke or industrial products, or contact with paint or soil [2,3,4]. Heavy metals are highly toxic through nerve and liver toxicity, bone deposition, and inhibiting vital enzymes in organisms [5]. Furthermore, because of their many biological differences from adults, fetuses are highly susceptible to teratogens such as heavy metals, even at low exposure levels that are generally harmless to their mothers [6]. Exposure to these metals has been identified as a potential risk factor for adverse effects on pregnant women and the developing fetus, including reproductive disorders, low birth weight, reduced birth length, lowered head and chest circumferences, and poor mental development [4]. During prenatal and early life developmental stages, fetuses and infants are unable to adequately detoxify and excrete heavy metals due to their immature immune systems, leading to their accumulation in the body [7]. Exposure during pregnancy, especially during organogenesis, the most vulnerable stage of human development due to the high degree of fetal cellular division and differentiation, may cause more detrimental effects, such as permanent structural and anatomical changes in the fetus [8]. Exposure to heavy metals during organogenesis can result in permanent structural and anatomical alterations. Alternatively, exposure that occurs after organogenesis can cause functional problems [9]. The placenta is generally recognized as a barrier that prevents the passage of harmful substances and protects the embryo and fetus from toxic exposure. Heavy metals such as Pb, Hg, and As can easily cross the placenta and accumulate in fetal tissues, while Cd can partially cross the placenta [10]. They potentially cause damage to developing fetal organs and systems [4,11].

The effects of metals on pregnancy and birth outcomes have been investigated [12,13,14] and reviewed in some studies [11,15,16,17]. Previous studies have linked single metal exposure with adverse birth outcomes. However, subsequent studies have examined multiple toxic metals and elements, providing a more accurate representation of real-world exposure. In the literature, studies have been conducted to determine the levels of various metals and elements in one or, rarely, two or more biological materials, such as maternal blood, urine, and cord blood [4,18,19]. Studies have reported associations of multiple metal and element exposures with pregnancy and birth outcomes such as birth weight, neonatal size, preterm birth, SGA, miscarriage, and placental characteristics [4,8,20].

The CDC has established recommended safe serum levels of Pb (5.0 μg/dL) and Hg (3.5 μg/L) for pregnant women, but these levels are not available for As and Cd [2]. Cadmium (Cd), a non-essential toxic element for humans, is released into the environment through various pathways, including smoking, combustion of fossil fuels, mining activities, sewage sludge, fertilizer application, and industrial waste discharge [21]. Cd exposure occurs through ingestion, inhalation, and dermal absorption; dietary intake and smoking are the primary routes for individuals without occupational exposure [7,22]. Cd tends to accumulate in various human organs, including kidneys, liver, lungs, and bones, with a long biological half-life of 10–30 years [23]. Cd has been classified as a Group I human carcinogen due to its carcinogenic properties [7]. Cd can cross the placental barrier and affect the fetus. In this way, intrauterine Cd exposure has been associated with adverse birth outcomes such as IUGR, preterm birth, and reduced head circumference [24,25]. Animal studies have shown that young animals exposed to Cd before birth have shown effects on behavior and learning [1]. In addition, Cd exposure during childhood is associated with higher body mass index (BMI), altered sexual characteristics in both males and females, cognitive dysfunction, and behavioral problems [7,26]. Furthermore, Cd exposure early in life is associated with an increased risk of chronic diseases later in life, including cardiovascular, renal, and neurological conditions, and some types of cancer [7,26]. As Cd accumulates in tobacco leaves, smoking is an important way of exposure that pregnant women should especially avoid. The CDC’s National Health and Nutrition Examination Survey found that the detection rate for urine, blood, and serum As, Pb, Hg, and Cd ranged from 83–99% among both pregnant and non-pregnant women in the US from 1999–2016, with higher mean urinary total As, urinary Hg, and urinary Pb levels in pregnant compared to non-pregnant women [27]. Kippler et al. showed an inverse relationship between Cd concentration in the placenta and zinc in cord blood, indicating that Cd may impair zinc transfer to the fetus [28].

Arsenic (As) exists in highly toxic inorganic and non-toxic organic forms. Inorganic As is found in soil and groundwater, so human exposure occurs primarily through drinking water, usually from untreated wells. Exposure to organic As occurs through seafood consumption and is not considered harmful to humans [29]. It can easily cross the placental barrier and affect the fetus. Studies have shown that pregnant women with high As exposure detected in urine and blood samples have a significantly higher likelihood of spontaneous abortion, preterm birth, fetal stillbirth, and congenital abnormalities [30,31]. A study in Wuhan reported that increased maternal urinary total As concentrations were significantly associated with decreased fetal birth length and birth weight [32]. There are several epidemiological studies that suggest that oral exposure to inorganic arsenic might increase the risk of congenital defects or abortion in exposed women. Fry et al. [33] found that in utero As exposure was associated with a fetal systemic inflammatory response due to increased proinflammatory cytokines and stress response proteins, leading to biological changes in signal transduction, cell adhesion, proliferation, and apoptosis. Inorganic As is considered a group 1 human carcinogen, and there are some data suggesting that it may increase the risk of transplacental cancer in humans [34].

Mercury (Hg) exposure is hazardous for pregnant women and their babies because mercury compounds can cross the placenta and the blood–brain barrier, potentially affecting the developing fetus as a developmental neurotoxin [35]. The neurological and renal systems are the most sensitive targets of mercury toxicity [1]. Animal studies have also identified cardiovascular, hematological, immunological, and reproductive toxic effects of mercury [1]. Hg exposure is thought to contribute to adverse birth outcomes due to mercury’s effect of inducing apoptosis, disrupting the endocrine system, and thus affecting reproductive hormones, and causing oxidative stress [36]. Hg exposure occurs predominantly through the consumption of contaminated fish. Still, it can also occur through inhalation of vapors from dental amalgams or industrial processes such as coal burning, mining, or waste incineration [37]. Hg is widely used in the health sector. Sphygmomanometers, thermometers, and certain gastrointestinal devices in a hospital may contain mercury [38]. If these thermometers and sphygmomanometers are broken, Hg can be released into the environment and inhaled, causing neurotoxicity [38]. For these reasons, the use of Hg thermometers and sphygmomanometers has been abandoned in many, but not all, health institutions, including our hospital. Hg can still be found in many areas of healthcare facilities, including fluorescent lamp ballasts, thermostat switches, and batteries [35,38,39]. While the confounding effect of fish consumption during pregnancy, which can provide omega-3 fatty acids and other nutrients essential for the developing fetus, increases the risk, there is also a potential adverse effect of Hg exposure during pregnancy on the fetus. In some studies, Hg levels in hair, maternal blood, or cord blood of women who gave birth preterm were significantly higher than those who gave birth at term and may be associated with preterm birth [37,40].

Lead (Pb) is an environmental contaminant whose widespread use has caused extensive environmental contamination and health impacts worldwide, and which can pass freely through the placenta [41]. Consequently, there is considerable concern about the potential adverse effects of prenatal Pb exposure on the developing fetus. Exposure to Pb during the fetal period and early life, even at low levels, is associated with preterm birth and small-for-gestational-age births [42,43]. Pb has been linked to several adverse health effects, including childhood growth retardation [21], impairment of cognitive and behavioral functions [44,45,46], hematopoietic, immunologic, renal, cardiovascular and reproductive system toxicity even in low levels [46,47]. Since Pb was phased out of gasoline in the 1970s, Pb levels in the general population have been reduced [48]. However, human exposure continues through contaminated dust and soil, drinking water, lead-based paint, cigarette smoke, byproducts of industrial processes, and ceramic glazes, among other sources. Numerous studies, typically by determining levels in cord blood or maternal blood, have demonstrated adverse effects of Pb exposure during pregnancy on prematurity, head circumference, birth weight, and ponderal index, more frequently in male fetuses [49,50,51].

Studies have shown that exposure to Pb and As may be negatively associated with birth weight, and they also negatively affect birth length and head circumference [52].

A recent study in North Carolina assessed exposure to Cd, Hg, Pb, and As and found detectable levels of each metal in more than 55% of pregnant women; some women had blood levels exceeding the 95th percentile for the US population [2].

While prenatal exposure to heavy metals has been well studied over the past few decades, information on threats to the fetus at low exposure levels is either limited or inconsistent.

Many trace elements are essential for humans, but toxicity can occur when the upper tolerable level is exceeded [53]. Manganese (Mn), copper (Cu), and selenium (Se) are trace elements that belong to the same period of the Periodic Table, suggesting they have similar effects on biomolecules across various biological systems [54]. These elements play an essential role in fetal cell growth [55]. On the other hand, an overload of these trace elements can cause toxicity to the fetus; for example, Mn [56] and Se [57] can have adverse effects when present at high levels.

Manganese (Mn)is an essential mineral nutrient that plays a vital role in fetal development and other important aspects of metabolism. The general population is exposed to manganese through consumption of food and water, inhalation of air, and dermal contact with air, water, soil, and consumer products that contain manganese [1]. High manganese can cause potential neurotoxic effects, especially in infants [58]. Dai et al. demonstrated that prenatal Mn exposure was positively associated with the ponderal index (PI) and negatively associated with physical growth in childhood, with the greatest impact during early life [59]. Excessive maternal and fetal Mn exposure may have adverse effects on neuromotor function in children up to 3 years of age [60].

Selenium (Se) is a trace element that plays a crucial role in various human biochemical functions, particularly in antioxidant systems [61]. The recommended daily Se intake is 50–200 μg. Although Se is an essential nutrient, exposure to high levels through inhalation or ingestion can cause adverse health effects. The primary routes of exposure are, in decreasing relative proportions, breast milk, food (abundant in sea fish), water, and air [62]. Se is transferred to fetuses through the placenta [63,64]. In the Czech Republic, Se was detected in infant cord blood (4.0–82.6 µg/L) [65], in fetal tissues (2.8 µg/g) [66], and in the blood of children (5.0–98.2 µg/L) [65].

Copper (Cu), an essential micronutrient, has a beneficial effect on neurodevelopment in children; however, overexposure can have a toxic impact on neurodevelopmental outcomes [67]. In a Spanish birth cohort, maternal serum Cu concentrations in the first trimester of pregnancy were associated with lower motor scores in children [68]. In a recent Norwegian birth cohort study, second-trimester blood Cu levels were positively associated with the risk of cerebral palsy in children [69]. Evidence from animal experiments indicates that excessive levels of Cu can reduce motor function and trigger oxidative stress [70,71]. Low-dose Cu exposure can impair motor function, alter protein levels involved in mitochondrial function and neurodevelopment, and increase reactive oxygen species production [72]. A multi-country meta-analysis found significant positive associations between maternal Cu and preterm birth risk and negative associations with gestational duration [73]. In a case–control study of neural tube defect cases, serum Cu and whole blood Pb levels were significantly higher than in controls [74].

Trace element levels in newborns are influenced by many factors, including maternal health, birth history, lifestyle, diet, and maternal accumulation of trace elements [75]. In the literature, there is little data on the exposure of preterm infants to heavy metals and trace elements in the NICU.

In the NICU, neonates, especially preterms, are vulnerable to toxic chemical exposure because of their extreme anatomical and physiological immaturity. In particular, preterm infants have increased vulnerability to neurotoxic agents such as heavy metals due to the immature blood–brain barrier and ongoing neurological developmental processes [76,77]. It is, therefore, critical to ensure that potential toxic chemical exposures are assessed [78,79]. In the literature, there are studies investigating the association of prenatal heavy metal and trace element exposure with adverse birth outcomes such as preterm birth and SGA [13,15,80,81,82]. There are few studies on the exposure to heavy metals and trace elements (Hg, Pb, Cd, As, Mn) regarding the treatments (TPN and/or blood transfusions) received by preterm infants in the NICU [83,84].

Preterm infants are particularly susceptible to environmental exposure to heavy metals and trace elements during the perinatal period. This study aimed to determine the concentrations of selected elements (Hg, Pb, Cd, As, Mn, Se, and Cu) in three biological matrices—maternal urine, neonatal urine, and cord blood—collected from preterm newborns and their mothers in the Neonatal Intensive Care Unit (NICU). In addition to assessing the interrelationships among element levels across these bio-matrices, we also examined their associations with maternal and neonatal characteristics, including maternal age, parity, gestational age, body mass index (BMI), pregnancy weight gain, birth weight percentile, head circumference percentile, anogenital distance (AGD), and stretched penile length (SPL). Few studies have simultaneously measured concentrations of these heavy metals and trace elements in such diverse samples—cord blood, maternal urine, and repetitive neonatal urine—of mother–preterm infant pairs.

This study will enable us to contribute to the literature on intrauterine and postnatal exposure levels of heavy metals and trace elements in preterm infants in the NICU. Furthermore, investigating the associated factors of exposure will enable further suggestions on measures that can be taken.

## 2. Materials and Methods

Preterm newborns born before 35 weeks of gestational age by normal vaginal delivery or cesarean section in Hacettepe University Faculty of Medicine Children’s Hospital between 1 August 2021, and 1 January 2022, and hospitalized in the NICU after birth, and their mothers older than 18 years of age were included in the study. Neonates with anencephaly and critical congenital anomalies, such as critical congenital heart defects that significantly reduce life expectancy, were excluded.

Informed consent was obtained from the mothers included in the study before delivery. A researcher (MC) conducted a face-to-face interview with the mother to complete the study file and collect information on demographic characteristics. The mother’s medical history was obtained from hospital records.

### 2.1. Working Protocol

At birth, the investigators collected 3 mL of cord blood samples from the umbilical cords of babies born in the delivery room. These samples were collected directly into glass test tubes containing depletion and heparin. The samples were centrifuged at 800× *g* for 15 min to obtain plasma.

A 3 mL urine sample was collected from the mother and her infant, hospitalized in the NICU, within the first 24 h after birth. The urine sample from the newborn was collected by squeezing sterile cotton gauze swabs placed in the diaper into glass tubes when the swabs were wet with urine. The mother’s urine sample was collected in a replaceable glass beaker. On the day the neonates were transferred from the NICU to the general care unit, a second 3 mL urine sample was collected from each neonate.

All samples were placed in glass tubes and bottles. All serum and urine samples were aliquoted and stored at −80 °C until analysis.

The newborn’s medical history was obtained from hospital records. Within 24 h after birth, all newborns underwent general physical examinations performed by the same pediatrician (MC) to minimize measurement errors. In addition, anthropometric measurements (birth weight, head circumference), anogenital distance (AGD), and penile measurements [stretched penile length (SPL)] were performed for all newborns. AGD was measured following the methodology described by Salazar-Martinez et al. [85]. SPL was measured with a rigid plastic ruler from the tip of the glans to the pubopenile skin junction. At the same time, tension was applied to ensure maximum stretching of the penis [86].

During labor and the monitoring period in the NICU, all medical devices, procedures, supplies, treatments [e.g., umbilical catheters, central catheters, total parenteral nutrition (TPN), intravenous fluids, nasogastric (NG)/orogastric (OG) catheters, urinary catheters, intubation tubes, tracheostomy cannulas, nasal Continuous Positive Airway Pressure (nCPAP), bonnet, blood product transfusions] and infant feeding methods [breastfeeding, formula, NG/OG catheters] that could be potential sources of heavy metal and trace element exposure were recorded in the study file.

### 2.2. Measurement of Heavy Metals and Trace Elements

Deionized water (18.2 MΩ·cm) was obtained using a Thermo Scientific Barnstead Micropure Water Purification System (Waltham, MA, USA).

Nitric acid, hydrogen peroxide, hydrochloric acid, and single-element standard solutions (1000 µg/L each for all elements studied) were obtained. The levels of Pb, As, Cd, Hg, Mn, Se, Cu, and Zn were measured by Inductively Coupled Plasma—Mass Spectrometry (ICP-MS, iCAP RQ ICP-MS, Thermo Fischer Scientific, Waltham, MA, USA). A CEM MARS™ 6 one-touch microwave digestion system (CEM Corporation, Matthews, NC, USA) was used for microwave digestion. The autosampler was from Teledyne CETAC Technologies (Omaha, NE, USA).

Deionized water (2 mL), nitric acid (2 mL), hydrogen peroxide (1 mL), and hydrochloric acid (0.2 mL) were added to the sample and left for 10 min for pre-digestion in a fume hood. The microwave digestion process was initiated with a specific temperature program (Ramp time: 40 min, Hold time: 30 min; 190 °C, 1500 W). Samples (100 µL) were transferred to a pre-cleaned, dry microwave digestion dish. Gold (200 µg/L) was added to all calibration and rinse solutions to enhance mercury washout and minimize memory effects. After digestion, the rotor was maintained at 25 °C for 15 min, and the microwave digestion vessels were cooled and placed in a fume cupboard. The digested sample solution was quantitatively transferred to a pre-cleaned volumetric flask, rinsed with deionized water, and then rinsed again. Calibration standards were prepared by serial dilution of mixed working standards to cover the appropriate concentration ranges for linearity assessment.

*Internal standards (ISs):* IV-ICPMS-71E (Sc, Ga, In, Tl, Bi) plus IV-ICP-Ge-10 (1000 µg/mL Ge single element) were used together to prepare the internal standard mix. The ISs [bismuth (Bi), germanium (Ge), rhodium (Rh)] were added from a stock solution containing 10 mg/L of each element (final concentration is 20 μg/L), and the total volume was adjusted to a certain volume with deionized water. The prepared sample solutions were vortexed at 2500 rpm by using a test tube vortex mixer for 30 s. A procedural blank was prepared without a sample matrix. The monitored isotopes were lead (208Pb, IS 209Bi), mercury (202Hg, IS 205Tl), arsenic (75As, IS 72Ge), cadmium (111Cd, IS 115In) manganese (55, 45Sc IS), selenium (76Se, IS 72Ge), copper (65Cu, 45Sc IS) (mass).

*Instrument Parameters:* Plasma power (RF) was 1600 W; plasma gas was argon (Ar, 15.0 L/min); nebulizer gas was used at 0.9 L/min; auxiliary (aux) gas was used at 0.6 L/min. Standard quartz torch with appropriate injector (1.0–2.0 mm i.d.) was used. Helium (He) gas flow (cell) was started at 5 mL/min. Autosampler rinse was 90 sec as Hg was measured. Dwell time per isotope is between 50 and 200 msec.

*Linearity:* The linearity is demonstrated using a six-point calibration curve. Calculations (in µg/L) were made against intensity [cps]10^3^. The mean R^2^ value for ten independent experiments for the elements was: 0.998 for Pb, 0.999 for As, 0.999 for Cd, 0.993 for Hg, 0.998 for Mn, 0.997 for Se, and 0.998 for Cu.

*Limit of Detection (LOD) and Limit of Quantification (LOQ):* The LOD values for the measured elements were: 0.0009 µg/L % for Pb, 0.0009 µg/L for As, 0.0008 µg/L for Cd, 0.001 µg/L for Hg, 0.0009 µg/L for Mn, 0.0008 µg/L for Se, and 0.0007 µg/L for Cu. The LOQ values for the measured elements were: 0.0021 µg/L % for Pb, 0.0028 µg/L for As, 0.0024 µg/L for Cd, 0.0032 µg/L for Hg, 0.0025 µg/L for Mn, 0.0026 µg/L for Se, and 0.0022 µg/L for Cu.

*Accuracy, Reliability, and Traceability:* Recovery from Certified Reference Material (CRM) was 98.9% for Pb, 92.8% for As, 101.8% for Cd, 89.7% for Hg, 99.7% for Mn, 99.4% for Se, and 97.6% for Cu.

*Precision:* Relative Standard Deviations (RSDs) for the measured elements for ten replicates were as follows: 5.2% for Pb, 6.1% for As, 2.3% for Cd, 6.7% for Hg, 2.4% for Mn, 2.8% for Se, and 3.4% for Cu.

*Sensitivity:* The background equivalent concentrations (BECs) for the measured elements were as follows: 0.0029 µg/L/for Pb, 0.031 µg/L for As, 0.0012 µg/L for Cd, 0.029 µg/L for Hg, 0.009 µg/L for Mn, 0.004 µg/L for Se, and 0.006 µg/L for Cu.

### 2.3. Statistical Analysis

All statistical analyses were performed using IBM SPSS Statistics version 23.0 (IBM Corp., Armonk, NY, USA). The distribution of continuous variables was assessed using the Shapiro–Wilk test and visual inspection of histograms and Q–Q plots. Continuous variables were expressed as mean ± standard deviation (SD) or median [25th–75th percentile], depending on the normality of distribution, whereas categorical variables were presented as frequencies and percentages. Elemental concentrations below the limit of detection (LOD) were replaced with LOD/2 for statistical calculations. A two-tailed *p*-value < 0.05 was considered statistically significant.

Differences between the first and second neonatal urine samples (Nu1 vs. Nu2) were analyzed using the paired samples *t*-test for normally distributed data or the Wilcoxon signed-rank test for non-normally distributed data.

Pairwise correlations of element concentrations between biological matrices (Mu & Cord, Mu & Nu1, Cord & Nu1, and Nu1 & Nu2) were examined according to data distribution using either Pearson’s correlation for normally distributed variables or Spearman’s rank correlation for non-normally distributed variables.

Correlations between maternal and neonatal characteristics (e.g., maternal age, parity, gestational week, birth weight percentile, anogenital distance, and stretched penile length) and element concentrations in different matrices were also evaluated using Pearson’s or Spearman’s correlation coefficients, as appropriate.

## 3. Results

### 3.1. Samples Obtained from Participants

Among 48 participant mothers and 58 neonates recruited, 40 mother–newborn pairs having all four appropriate samples of cord blood, Mu, and first and second Nu samples were included in the analysis. Among 48 mothers, 25 were multiparous, and 15 were primiparous.

Among the newborns in our study, 9 were twins (*n* = 18), 1 was a triplet (*n* = 3), and 37 were singletons. 27 neonates out of 37 singletons and 13 out of 21 multiple births with complete and appropriate samples were included. Only one newborn born from each multiple pregnancy was included in the study.

### 3.2. General Characteristics of Newborns and Exposures in NICU

Forty preterm neonates born before 35 weeks and their mothers were included in the study. The mean (± SD) (min–max) gestational duration was 31.9 ± 2.0 (27.6–34.5) weeks, and 62.5% were male. Birth weight percentile was mean (± SD) 40.2 (±28.5), and head circumference was 52.5 (±28.8)cm. Twenty-five (62.5%) of the newborns were male. The mean (± SD) anogenital distance measured immediately after birth was 1.78 (±0.41) cm, and the stretched penile length was 2.36 (±0.33) cm. Fifteen (37.5%) babies were first-born, and 25 (62.5%) were later-born. Of all newborns, 22 (55.0%) were formula-fed, and 27 (67.5%) received TPN (Table 1).

The median [25–75 percentile] duration of NICU hospitalization was 7.0 [4.0–9.8] days. The duration of breast milk intake was a median of 7.0 (5.0–10.8) days, and formula intake was 1.0 [0.0–6.8] days. The duration of TPN administration was 3.5 [0.0–6.8] days. Only 6.9% (*n* = 4) of the patients received blood product transfusions.

### 3.3. Heavy Metal and Trace Element Levels in Different Biomatrices

**Figure 1:**
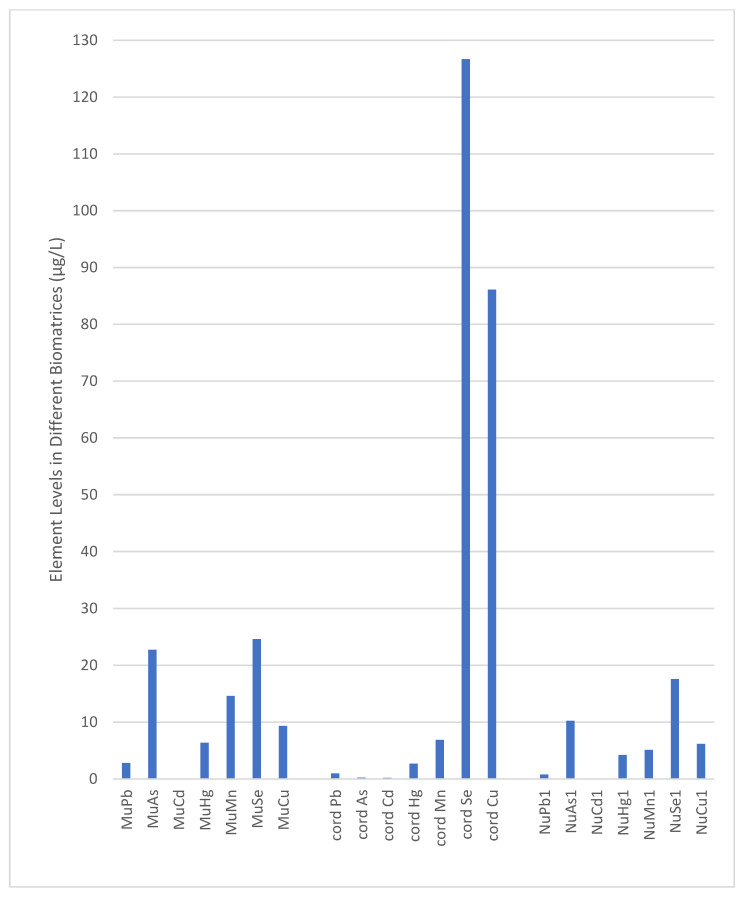
Median Element Levels According to Biomatrices (µg/L). Mu: Maternal urine; Nu: Neonatal urine; Pb: Lead; As: Arsenic; Cd: Cadmium; Hg: Mercury; Mn: Manganese; Se: Selenium; Cu: Copper.

The partition of essential minerals and environmental metals, as well as their associations between maternal and fetal umbilical cord blood, was metal-specific. Cord Pb, As, and Hg levels were found to be lower than in Mu (*p* < 0.001), while Se and Cu levels were higher (*p* < 0.001, Table 2, Figure 1). All elements’ excretion levels were higher in Mu than in Nu1 and Nu2 (*p* < 0.001, Table 2, Figure 1).

When comparing the elemental levels in cord blood and first neonatal urine, NuCd, Mn, Se, and Cu levels were lower, whereas As and Hg levels were significantly higher than those in cord blood. No difference was observed for Pb (Table 2, Figure 1).

Se had the highest cord transfer with a median cord Se level of 126.67 µg/L, followed by Cu with a median cord level of 86.11 µg/L among the elements studied. Se and As were the most excreted elements in maternal and infant urine (Table 2, Figure 1).

A positive correlation was found between maternal and the first neonatal urine Cu levels (r = 0.35). No other linear relationship was found when the correlation between heavy metals and trace elements in all three biomatrices (maternal urine, cord blood, and infant urine) was examined (Table 3).

A positive linear correlation was observed between the first and second infant urine levels of all elements studied. The lowest correlation was observed between the first and second infant urine levels of Cu and Cd (Table 3).

A significant increase in the excretion of all metals and elements in infant urine was observed over time (Table 4, Figure 1). The same significance was observed in the univariate analysis of variance adjusted for TPN and/or infant formula intake (*p* < 0.001).

### 3.4. Correlations Between Mother–Child Pair Characteristics and Element Levels

Maternal urinary Se levels increased with increasing maternal age (r_s_ = 0.41), but not with cord Se levels. Parity was positively correlated with maternal Cd and negatively correlated with Hg at low levels (Supplementary Table S1).

NuHg1 was negatively correlated with both penile length and anogenital distance. (r_s_ = −0.48, *p* = 0.016; r_s_ = −0.33, *p* = 0.040). NuHg1, and NuHg2 were negatively correlated with penile length (r_s_ = −0.48, *p* = 0.016; r_s_ = −0.58, *p* = 0.003) (Supplementary Tables S3 and S4).

In addition, both MuMn and cord Mn levels were moderately negatively correlated with penile length (r_s_ = −0.46, *p* = 0.020; r_s_ = −0.40, *p* = 0.048, respectively). (Supplementary Tables S1 and S2).

NuSe1, and cord Se were positively correlated with gestational week (r_s_ = 0.35, *p* = 0.027; r_s_ = 0.42, *p* = 0.008 (Supplementary Tables S2 and S3).

NuPb1 and NuPb2 were positively correlated with birth weight percentile (r_s_ = 0.36, *p* = 0.022; r_s_ = 0.38, *p* = 0.016) (Supplementary Tables S3 and S4). NuPb2 and MuSe correlated positively with maternal age (r_s_ = 0.33, *p* = 0.037; r_s_ = 0.41, *p* = 0.009) (Supplementary Tables S1 and S4).

MuHg was negatively (r_s_ = −0.36, *p* = 0.022), and MuCd was positively correlated with parity (r_s_ = 0.32, *p* = 0.044) (Supplementary Table S1).

## 4. Discussion

Our study, which examines exposure of mother–infant dyads to heavy metals and trace elements, fills a crucial gap in the existing literature. While some studies have explored this area, there is a dearth of evidence on the exposure of preterm neonates in the NICU to inorganic contaminants. In our research, we meticulously analyzed the concentrations of heavy metals and trace elements in urine samples from mothers and babies, and in cord blood from preterm infants hospitalized in the NICU. We then investigated their correlations with the characteristics of mother–baby pairs, shedding new light on this important area of study.

Currently, urine is considered the primary excretory medium for many mineral elements, reflecting recent exposure to these elements more accurately than other biomarkers such as hair and blood [87]. For example, urinary Se responds to exposure/intake over 24 h. Mn has a half-life of 10–42 days in the blood but less than 30 h in the urine [88]. Moreover, urine mineral levels reflect those in the body [88]. Therefore, we analyzed exposure to heavy metals and trace elements in this study using maternal and infant urine and cord blood.

### 4.1. Comparison of Cord Blood Levels

In our study, the median cord levels of Pb, As, Mn, and Cu were lower than the median levels reported in most literature studies [18,19,40,89,90,91,92,93,94,95,96,97,98]. Conversely, Cd and Se levels were approximately within established reference ranges [7,19].

In comparison to data from Bocca et al. [99], our median cord blood levels for As, Cd, Hg, Pb, and Mn were higher, except for Se, underscoring the substantial geographic and population heterogeneity in exposure profiles.

### 4.2. Comparison of Maternal Urine Levels

Our median maternal urine levels of As, Hg, Pb, Mn, and Se were higher than those reported by Bocca et al. [99] in Spain, except for Cu and Cd. Bocca et al. also reported that maternal urinary concentrations of As, Cu, Hg, and Se decreased during pregnancy, potentially leading to variations in fetal exposure [18]. In our study, we could not make such an observation because we only collected maternal urine at delivery.

In our study, median MuCu, MuMn, and MuSe concentrations were also higher than those reported by Guo et al. in China [100].

These differences may be related to differences in environmental exposures and dietary characteristics among mothers across geographies.

### 4.3. Placental Modulation of Heavy Metals

Our findings provide key insights into the transplacental transfer of heavy metals. We observed that cord levels of As, Hg, Pb, and Cd were significantly lower (*p* < 0.001) than those of other elements; and cord levels of As, Hg, and Pb were significantly lower than corresponding Mu levels (*p* ≤ 0.001). This suggests that the amount transferred from mother to baby is lower, and the placenta actively modulates the transfer rate of these elements. This supports the existing literature, which indicates that elements such as Hg and Pb exhibit free transplacental passage from the mother to the fetus [40], but that elements like Cd exhibit restricted transplacental passage [101,102,103].

### 4.4. Enhanced Transfer of Essential Trace Elements

In contrast to the heavy metals (Pb, As, and Hg), cord levels of Se and Cu were higher than Mu levels (*p* < 0.001 for Cu). In Sun H. et al.’s study [19], while the cord blood median Cd was lower, cord Se levels were higher than Mu levels. This may indicate a more prevalent maternal-to-infant transmission or selective fetal incorporation.

Specifically, Se and Cu exhibited the highest concentrations among all elements analyzed in cord blood. While Se transfer is considered limited [104,105], its high concentration likely reflects its storage in the fetal liver between 20 and 40 weeks of gestation [105] and the potential for increased maternal intake.

Except for Se, the findings of our study were different from the findings of some other studies that suggested [40,101,102,103] a high degree of maternal–fetal transfer of Hg, Pb, and Se for Hg and Pb, but not for As and Cd. The different findings of our study on this issue may be due to the fact that we studied a preterm population, in which physiologic characteristics may differ.

### 4.5. Correlations of Elemental Levels in Different Biomatrices

There was no linear correlation of heavy metals and trace elements in all three different biomatrices (Mu, cord blood, and infant urine) except for maternal and Nu Cu levels. Based on this result, we suspected that the exposure may have come from different sources that we were unable to demonstrate. While Mu mainly indicates maternal exposure, cord blood and the baby’s first urine reveal maternal exposure, and the second infant’s urine reveals NICU exposure. Due to the barrier function of the placenta, it can be thought that heavy metals and elements exposed to the mother do not pass to the baby through the cord blood at a level that affects the baby. The higher median excretion levels of all heavy metals and trace elements in Mu than in infant urine samples in our study may also support this result. Higher concentrations excreted through the Mu and the placental barrier may reduce neonatal exposure and urinary excretion.

### 4.6. Endocrine-Disrupting Effects on Genital Development

Disorders of penile development that include hypospadias and micropenis are among the most frequent male congenital disabilities worldwide [106]. The prevalence of micropenis, defined as penile length below the 2–2.5 SD range, varies significantly, ranging from 0.015 to 0.66% [107,108]. Despite the numerous unresolved questions, the impact of fetal exposure to endocrine-disrupting chemicals on the development of male penile disorders is now widely accepted. Hg and As are among the potent environmental estrogens defined as metalloestrogens (others: Cd, Co, Ni, Pb, etc.) [109]. They mimic the effects of estradiol by activating the Erα receptor [109].

In our study, we found a novel association between element exposure and male genital development. Both the first and second neonatal urine Hg levels (NuHg1 and NuHg2), along with maternal urine Mn levels (MuMn) and cord Mn levels, were negatively correlated with penile length. Furthermore, the first NuHg level, primarily reflecting prenatal exposure, showed a negative correlation with anogenital distance.

These results lend support to the theory of metallohormone effects, particularly for Hg, which acts as a metalloestrogen [101]. Prenatal Hg exposure has been suggested to be associated with adverse pregnancy outcomes due to Hg’s ability to induce apoptosis, disrupt the endocrine system, affect reproductive hormones, and induce oxidative stress [36,37,40].

The negative correlation involving both prenatal (NuHg1) and postnatal (NuHg2) markers suggests that the endocrine-disrupting effects of mercury may be exerted throughout this critical developmental window. Confirmation of these relationships requires larger-scale, prospective studies. To our knowledge, there are no studies in the literature on the effects of prenatal Hg exposure on penile length or anogenital distance in premature infants.

Mercury, which is known as a developmental neurotoxin, has been mostly shown in animal studies to have many toxic effects on nearly every organ system in the fetus, including the reproductive system, as we have observed in our study [1,35]. Pregnant women should be cautioned not to consume large deep-sea fish at high risk of contamination, which is the most important source of mercury exposure. In addition, they should also be warned about other possible sources of Hg, such as dental amalgams and inhalant exposure to industrial processes (coal burning, mining, or waste incineration) during pregnancy.

It has been suggested that Mn plays a fundamental role in the development of reproductive system [110]. Mn has been shown to increase LHRH at high levels, possibly associated with precocious puberty in both boys and girls [110,111]. A recent animal study reported that intrauterine Mn exposure of male rats caused oxidative damage and histological alterations in male reproductive organs, and disrupted sex hormone levels, ultimately impairing spermatogenesis and sperm quality in rats [112]. The negative association between maternal and cord Mn levels and penile length observed in our study may reflect the possible effect of intrauterine Mn exposure on the male reproductive system. However, to our knowledge, no studies in the literature have examined the effects of prenatal Mn exposure on penile length in humans.

Because of possible adverse effects on neurological development, growth, and the reproductive system at high prenatal exposure, pregnant women living, for example, near mining areas should be warned about water and food sources that may be contaminated with Mn, soil, and consumer products that may contain Mn.

### 4.7. Lead Levels and Birth Weight

We found that the first and second infant urine Pb levels (NuPb1, NuPb2) were positively correlated with birth weight percentile. Several studies have examined the effects of heavy metal exposure on newborn anthropometrics [19,36,113,114]. Since heavy metals cause physiological immaturity during pregnancy and early life, they can pose a serious threat to fetal and infant health [26]. Among these, Cd was previously found to have the most significant impact on several anthropometric birth outcomes [36], but we did not observe any correlations in our study.

Our results were similar to those of Zinia et al., who demonstrated that cord blood Pb level was positively associated with birth weight [114], but they contradict the more prevalent literature demonstrating an inverse or null association [113,115,116,117]. This unexpected finding may be due to the influence of other factors, such as maternal nutrition, which can impact birth weight. As such, nutritional intake was not considered in this study, although it may have impacted birth weight [80]. Therefore, we could not completely exclude the influence of dietary intake on metal exposure measurements [118].

The health effects of intrauterine Pb exposure are diverse and associated with toxicity to every organ system, including anthropometric measures at birth. Neurological effects of Pb are of most significant concern because they are observed in infants and children; furthermore, these effects may result in lifelong decrements in neurological function. Therefore, pregnant women should be warned to avoid sources of lead exposure such as soil, water, lead-based paints, ceramic glazes, and especially cigarette smoke.

### 4.8. Selenium Levels and Gestational Duration

The average cord blood Se level is reported to be 35–107 μg/L in the literature, which is influenced by soil Se content in different geographic regions, gestational age, and serum Se concentration after 36 weeks [104,119]. In Nazemi et al.’s [104] study, cord blood Se levels of low birth weight babies with a gestational age of 28.82 ± 13.66 years were 77.32 ± 26.12 (μg/mL), which is considerably lower than our study (126.67 µg/L).

The positive correlation we found between Se levels (NuSe and cord Se) and gestational age in premature infants is consistent with a prospective study [120] and an international meta-analysis [121]. Bebars et al. [120] demonstrated significant positive correlations between gestational age and umbilical cord Se levels and found a significantly higher Se level in umbilical cord blood in newborns at term than preterm (72.25 ± 10.5 μg/L vs. 64.85 ± 7.67 μg/L) [120]. These values are nearly 50% lower than the median cord blood Se levels in our study (126.67 μg/L).

Monangi et al.’s [121] multi-national meta-analysis showed that maternal Se levels varied substantially across different sites. They showed that maternal serum Se concentration was positively associated with gestational duration [121]. We did not collect maternal blood. However, cord Se levels associated with maternal transmission are higher than those reported in the literature and are also associated with gestational duration, supporting the findings of this study.

The notably high median cord Se level in our preterm cohort (126.67 μg/L) suggests a potential protective role of high Se status against the oxidative stress inherent to prematurity [122]. This is likely influenced by site-specific factors, including maternal diet or supplementation, socioeconomic status, or local factors that might interact with Se levels and influence prematurity risk.

Blood Se level is associated with glutathione peroxidase (GPx) activity, which enhances antioxidative protection of the placenta and indirectly influences fetal growth, leading to intrauterine growth restriction (IUGR) at low levels [122]. Although hypothetical, the notably high cord Se levels in our preterm study group may have served as a protective mechanism against the oxidative stress to which preterm infants are highly exposed.

Se levels may depend on various factors, such as diet, supplementation, age, gender, geographic location, or genes [121,122]. Therefore, our study results may be related to maternal high Se intake through their diet or nutritional supplements, such as vitamins. However, we did not examine the mothers’ dietary or supplement intake in our study.

### 4.9. Selenium and Maternal Age

In our study, maternal urinary Se increased with increasing maternal age but not with cord Se levels. Similarly, Guo et al. [100] found that urinary Se levels in pregnant urine increased with age [100]. A study of pregnant women from Spain also found a positive relation between urinary Se and women’s age [87]. Positive correlations between age and serum Se [105,123,124] levels have also been reported.

### 4.10. Correlation of Maternal Urine Selenium and Copper Levels

We found a significant correlation between MuCu and MuSe in our study. Several previous studies have also reported correlations among these elements in pregnant women. Guo et al. reported significantly positive correlations among maternal urinary levels of Ca (Calcium), Fe (Iron), Cu, and Se, except Mn [100]. Lozano et al. reported a moderate positive correlation between maternal urinary Se and Cu (r = 0.27) [87]. Our findings corroborate this relationship, showing a similar correlation between maternal urinary Cu and Se.

The consistency of the Cu and Se correlation suggests that these two elements may share common sources of exposure or predictive factors in this population. These sources may be the diet or supplements used by the pregnant woman, which were not recorded in our study.

### 4.11. Postnatal Exposure in the NICU

A critical observation was the significant increase in the urinary excretion of *all* elements in infants’ urine at discharge (Nu2) compared with admission (Nu1). This effect persisted even after statistically adjusting for TPN and/or formula intake during NICU stay. This pattern suggests that the elevated excretion near discharge may be directly attributable to increased postnatal exposure to trace elements and metals in the NICU environment, rather than solely to residual in utero exposure. We propose that the increased urinary output towards discharge could be a compensatory clearance mechanism aimed at reducing the overall body burden. This contrasts with the findings of Al-Saleh et al. [90], who reported a decrease in blood Hg, Pb, and Mn levels at discharge in a similar cohort of preterm neonates. They also suggested that preterm newborns may be exposed to high levels of metals in utero [90]. We did not analyze the blood levels of the newborns at discharge in our study. The difference highlights the necessity for further research to distinguish between increased NICU exposure and altered postnatal clearance mechanisms. Close monitoring of materials that may be contaminated with toxic elements, such as water, TPN, formula, or blood products used in NICUs where vulnerable populations, especially preterm neonates, are hospitalized, and additional exposure studies could provide more information and reduce the risk of exposure.

### 4.12. Strengths and Limitations

Heavy metal and trace element levels in three different biomatrices—cord blood, Mu, and Nu—were measured prenatally and in the early postnatal period in preterm infants under 35 weeks of age who were hospitalized in the NICU. Furthermore, to our knowledge, no other study in the literature has simultaneously measured heavy metals and trace elements in these three biomatrices, performed biphasic measurements, and followed up on infant urine, as in our study.

In this study, valuable information on maternal exposure of preterm infants to heavy metals and trace elements through cord blood and in the NICU environment was obtained and contributed to the literature.

We did not collect blood samples from mothers and newborns at discharge from the NICU in this study, which would have facilitated comparisons with cord blood metal levels in our research and those reported in the literature. We preferred to take cord blood and urine from mothers and infants as a non-invasive method.

Due to the single-centre nature of our study and the relatively small sample size of 40 mother–infant pairs, advanced analysis could not be performed, thereby reducing the statistical power and generalisability of the results.

In this study, multi-element analysis was not performed. Further studies with a large sample size are necessary for the evaluation of multi-element interaction and confounding factors.

We did not investigate term infants as a control group for comparison of exposure. Our study population consists only of preterm newborns; therefore, we cannot compare their results with those of term infants.

Some potential confounders, such as maternal diet, nutritional supplementation, and environmental exposure history, which may influence heavy metal and trace element levels, were not recorded.

The instruments used in the NICU, as well as the formula and breast milk the infant receives, were not analyzed for heavy metals and trace elements. In this case, the effect of the procedures and nutrition on the baby’s urinary metal levels was not demonstrated.

## 5. Conclusions

Compared to maternal urine, cord blood had lower levels of Pb, As, and Hg, but higher levels of Se and Cu. This suggests that the placental barrier provides protection against some heavy metals.

The excretion of all elements increased in infant urine over time, possibly due to exposure to TPN or formula in the NICU rather than in utero exposure.

Se and Cu showed the highest cord transition, suggesting a protective mechanism against oxidative stress in preterm infants.

The negative correlations found in our study between maternal urine Mn, cord Mn levels, and NuHg1 levels with penile length or anogenital distance reveal endocrine-disrupting effects of these elements that have not been previously reported in the literature to our knowledge.

Our study contributes to the literature by suggesting transplacental transmission, presence, and exposure to some heavy metals and trace elements across different biomatrices in preterm infants. However, there is a need for more large-scale studies on the mechanisms of these exposures.

We believe that our study will increase awareness among healthcare personnel who care for pregnant women and preterm infants about the endocrine-disrupting effects of certain heavy metals and trace elements, and can help them make prevention recommendations.

## Figures and Tables

**Table 1:** General Characteristics of Mothers and Newborns.

Mother (*N* = 40)	Mean ± SD (Min–Max)
Mother’s age, years	29.9 ± 5.2 (19–39)
Mother’s height	162.7 ± 5.8
Mother’s BMI (preconceptional)	25.4 ± 5.0
**Pregnancy and Birth**	
Parity	2.3 ± 1.1 (1–5)
Birth order, 1st	15 (32.5)
Twin pregnancy	13 (32.5)
**Newborn (** ***N* = 40)**	
Gestational duration, weeks	31.9 ± 2.0 (27.6–34.5)
Sex, male	25 (62.5)
Birth weight, percentile	40.2 ± 28.5
Head circumference, percentile	52.5 ± 28.8
Anogenital distance, cm	1.78 ± 0.41
Stretched penile length (*n* = 25), cm	2.36 ± 0.33
TPN use	27 (67.5)
Formula intake	22 (55.0)

Mean ± SD (min–max); median [25–75 p]; *n* (%); SD: Standard deviation; BMI: Body Mass Index, TPN: Total Parenteral Nutrition.

**Table 2:** Heavy Metal and Trace Element Levels in Different Biomatrices (µg/L).

	GM	Mean	SD	Min	25 p	50 p	75 p	90 p	Max
*Pb*									
MuPb	2.80	2.91	0.81	1.41	2.36	2.81	3.41	4.08	4.78
Cord Pb	0.85	1.00	0.54	0.21	0.60	0.98	1.30	1.55	2.65
NuPb1	0.80	0.83	0.23	0.33	0.72	0.78	0.96	1.12	1.41
NuPb2	0.86	0.90	0.26	0.41	0.75	0.88	1.04	1.26	1.56
*As*									
MuAs	21.85	22.33	4.48	14.27	18.77	22.76	26.15	27.44	31.04
CordAs	0.24	0.26	0.13	0.11	0.17	0.23	0.32	0.45	0.63
NuAs1	9.97	10.23	2.37	6.11	8.29	10.24	11.24	14.50	16.20
NuAs2	10.54	10.76	2.22	7.52	8.94	10.26	12.34	14.13	16.23
*Cd*									
MuCd	0.12	0.13	0.03	0.08	0.11	0.12	0.14	0.17	0.25
Cord Cd	0.17	0.18	0.07	0.10	0.13	0.17	0.23	0.31	0.41
NuCd1	0.08	0.09	0.02	0.05	0.07	0.08	0.10	0.12	0.16
NuCd2	0.09	0.09	0.03	0.02	0.08	0.09	0.12	0.13	0.14
*Hg*									
MuHg	6.01	6.26	1.77	3.28	4.65	6.38	7.36	8.51	10.62
Cord Hg	2.74	3.13	1.68	1.08	1.96	2.67	4.27	5.40	8.77
NuHg1	4.27	4.35	0.86	3.01	3.67	4.20	4.76	5.98	6.37
NuHg2	4.86	4.98	1.13	3.24	4.18	4.95	5.67	6.79	7.99
*Mn*									
MuMn	11.38	12.37	4.76	4.24	7.62	14.61	15.81	17.50	21.18
Cord Mn	6.63	6.76	1.31	4.21	5.78	6.86	7.97	8.27	9.21
NuMn1	5.02	5.26	1.59	2.33	4.02	5.10	6.30	7.45	8.45
NuMn2	5.65	5.86	1.54	2.36	4.89	5.86	7.02	8.08	9.06
*Se*									
MuSe	24.43	24.55	2.42	19.87	22.43	24.61	26.43	27.45	28.17
Cord Se	121.43	123.25	20.61	78.56	108.98	126.67	140.21	149.88	156.71
NuSe1	17.63	17.82	2.67	13.21	15.65	17.58	20.04	21.72	24.12
NuSe2	18.57	18.86	3.45	13.27	16.29	18.18	21.28	23.60	28.75
*Cu*									
MuCu	9.47	9.81	2.74	5.66	7.83	9.35	10.87	14.49	17.45
Cord Cu	86.97	87.49	9.76	70.54	79.66	86.11	94.45	100.15	110.21
NuCu1	5.75	5.93	1.35	2.23	4.94	6.15	6.98	7.45	8.54
NuCu2	6.14	6.31	1.40	2.65	5.34	6.25	7.47	7.83	9.02

*N* = 40; Mu: Maternal urine; Nu: Neonatal urine; GM: Geometric Mean; SD: Standard Deviation; 50 p: Median; Min: Minimum; Max: Maximum.

**Table 3:** Correlation of Heavy Metals and Trace Elements Between Biological Matrices.

		Mu & Cord	Mu & Nu1	Cord & Nu1	Nu1 & Nu2
Pb *	r_s_	0.08	0.11	−0.01	0.94
	*p*	0.627	0.485	0.969	**<0.001**
As **	r_s_	−0.02	0.09	0.22	0.68
	*p*	0.928	0.583	0.171	**<0.001**
Cd **	r_s_	−0.29	−0.11	−0.09	0.52
	*p*	0.074	0.509	0.573	**0.001**
Hg **	r_s_	−0.19	−0.17	−0.20	0.85
	*p*	0.254	0.298	0.210	**<0.001**
Mn *	r_s_	0.13	0.07	−0.05	0.81
	*p*	0.421	0.666	0.782	**<0.001**
Se *	r_s_	−0.04	−0.20	0.21	0.73
	*p*	0.831	0.224	0.201	**<0.001**
Cu *	r_s_	−0.07	0.35	0.04	0.65
	*p*	0.669	0.026	0.789	**<0.001**

* Pearson correlation; ** spearman correlation; *p*: significance (<0.05); r_s_: Spearman correlation coefficient. Mu: Maternal urine; Nu: Neonatal urine; Pb: Lead; As: Arsenic; Cd: Cadmium; Hg: Mercury; Mn: Manganese; Se: Selenium; Cu: Copper.

**Table 4:** Changes in Heavy Metal and Trace Element Levels in Neonatal Urine Over Time, µg/L *.

	Nu1	Nu2	Sign ^&^
Pb	0.82 ± 0.23	0.90 ± 0.26	**<0.001**
As	10.24 [8.28–11.24]	10.26 [8.93–12.33]	**0.003**
Cd	0.08 [0.07–0.10]	0.09 [0.08–0.12]	**0.024**
Hg	4.19 [3.67–4.75]	4.95 [4.18–5.67]	**<0.001**
Mn	5.26 ± 1.59	5.85 ± 1.54	**<0.001**
Se	17.81 ± 2.67	18.86 ± 3.45	**0.008**
Cu	5.93 ± 1.35	6.31 ± 1.40	**0.043**

* Values are given as mean ± SD or median [25–75 p], ^&^ Paired sample *t* test or Wilcoxon test; Sign: significance; *N* = 40; Nu1: First neonatal urine; Nu2: Second neonatal urine; Pb: Lead; As: Arsenic; Cd: Cadmium; Hg: Mercury; Mn: Manganese; Se: Selenium; Cu: Copper.

## Data Availability

The data presented in this study are available on request from the corresponding author due to ethical considerations.

## Supplementary Materials

The following supporting information can be downloaded at: https://www.mdpi.com/article/10.3390/toxics13100898/s1, Table S1: Correlations of Maternal Urinary Heavy Metal and Trace Element Levels with Mother–Infant Characteristics; Table S2: Correlations of Cord Blood Heavy Metal and Trace Element Levels with Mother–Infant Characteristics; Table S3: Correlations of First Neonatal Urinary Heavy Metal and Trace Element Levels with Mother–Infant Characteristics; Table S4: Correlations of Second Neonatal Urinary Heavy Metal and Trace Element Levels with Mother–Infant Characteristics.

## Abbreviations

The following abbreviations are used in this manuscript:

NICU	Neonatal Intensive Care Unit
Hg	Mercury
Pb	Lead
Cd	Cadmium
As	Arsenic
Mn	Manganese
Se	Selenium
Cu	Copper
ICP-MS	Inductively Coupled Plasma—Mass Spectrometer
Mu	Maternal urine
Nu	Neonatal urine
SPL	Stretched Penile Length
AGD	Anogenital Distance
TPN	Total Parenteral Nutrition
NG	Nasogastric
OG	Orogastric
nCPAP	nasal Continuous Positive Airway Pressure

## References

[1] Centers for Disease Control and Prevention (CDC), Agency for Toxic Substances and Disease Registry ATSDR’s Substance Priority List. https://www.atsdr.cdc.gov.

[2] Sanders A.P., Flood K., Chiang S., Herring A.H., Wolf L., Fry R.C. (2012). Towards prenatal biomonitoring in North Carolina: Assessing arsenic, cadmium, mercury, and lead levels in pregnant women. PLoS ONE.

[3] Liu X.W., Hu Q.Y., Fang Z., Zhang X.J., Zhang B.B. (2009). Magnetic Chitosan Nanocomposites: A Useful Recyclable Tool for Heavy Metal Ion Removal. Langmuir.

[4] Issah I., Duah M.S., Arko-Mensah J., Bawua S.A., Agyekum T.P., Fobil J.N. (2024). Exposure to metal mixtures and adverse pregnancy and birth outcomes: A systematic review. Sci. Total Environ..

[5] Sanyal A., Rautaray D., Bansal V., Ahmad A., Sastry M. (2005). Heavy-metal remediation by a fungus as a means of production of lead and cadmium carbonate crystals. Langmuir.

[6] Wells P.G., Lee C.J., McCallum G.P., Perstin J., Harper P.A. (2010). Receptor- and reactive intermediate-mediated mechanisms of teratogenesis. Handbook of Experimental Pharmacology.

[7] Huang N., Wang B., Liu S., Wang K., Wang R., Liu F., Chen C. (2025). Cadmium exposure in infants and children: Toxicity, health effects, dietary risk assessment and mitigation strategies. Crit. Rev. Food Sci. Nutr..

[8] Huang K., Li H., Zhang B., Zheng T., Li Y., Zhou A., Du X., Pan X., Yang J., Wu C. (2017). Prenatal cadmium exposure and preterm low birth weight in China. J. Expo. Sci. Environ. Epidemiol..

[9] Caserta D., Graziano A., Lo Monte G., Bordi G., Moscarini M. (2013). Heavy metals and placental fetal-maternal barrier: A mini-review on the major concerns. Eur. Rev. Med. Pharmacol. Sci..

[10] Iyengar G.V., Rapp A. (2001). Human placenta as a ‘dual’ biomarker for monitoring fetal and maternal environment with special reference to potentially toxic trace elements. Part 3: Toxic trace elements in placenta and placenta as a biomarker for these elements. Sci. Total Environ..

[11] Amegah A.K., Sewor C., Jaakkola J.J.K. (2021). Cadmium exposure and risk of adverse pregnancy and birth outcomes: A systematic review and dose-response meta-analysis of cohort and cohort-based case-control studies. J. Expo. Sci. Environ. Epidemiol..

[12] Sun X., Liu W., Zhang B., Shen X., Hu C., Chen X., Jin S., Jiang Y., Liu H., Cao Z. (2019). Maternal Heavy Metal Exposure, Thyroid Hormones, and Birth Outcomes: A Prospective Cohort Study. J. Clin. Endocrinol. Metab..

[13] Wang Z., Huang S., Zhang W., Zeng X., Chu C., Li Q., Cui X., Wu Q., Dong G., Huang J. (2022). Chemical element concentrations in cord whole blood and the risk of preterm birth for pregnant women in Guangdong, China. Ecotoxicol. Environ. Saf..

[14] Wai K.M., Mar O., Kosaka S., Umemura M., Watanabe C. (2017). Prenatal Heavy Metal Exposure and Adverse Birth Outcomes in Myanmar: A Birth-Cohort Study. Int. J. Environ. Res. Public. Health.

[15] Khanam R., Kumar I., Oladapo-Shittu O., Twose C., Islam A.A., Biswal S.S., Raqib R., Baqui A.H. (2021). Prenatal Environmental Metal Exposure and Preterm Birth: A Scoping Review. Int. J. Environ. Res. Public. Health.

[16] Quansah R., Armah F.A., Essumang D.K., Luginaah I., Clarke E., Marfoh K., Cobbina S.J., Nketiah-Amponsah E., Namujju P.B., Obiri S. (2015). Association of arsenic with adverse pregnancy outcomes/infant mortality: A systematic review and meta-analysis. Environ. Health Perspect..

[17] Milton A.H., Hussain S., Akter S., Rahman M., Mouly T.A., Mitchell K. (2017). A Review of the Effects of Chronic Arsenic Exposure on Adverse Pregnancy Outcomes. Int. J. Environ. Res. Public. Health.

[18] Bocca B., Ruggieri F., Pino A., Rovira J., Calamandrei G., Mirabella F., Martinez M.A., Domingo J.L., Alimonti A., Schuhmacher M. (2020). Human biomonitoring to evaluate exposure to toxic and essential trace elements during pregnancy. Part B: Predictors of exposure. Environ. Res..

[19] Sun H., Chen W., Wang D., Jin Y., Chen X., Xu Y. (2014). The effects of prenatal exposure to low-level cadmium, lead and selenium on birth outcomes. Chemosphere.

[20] Tsuzuki S., Morimoto N., Hosokawa S., Matsushita T. (2013). Associations of maternal and neonatal serum trace element concentrations with neonatal birth weight. PLoS ONE.

[21] Afeiche M., Peterson K.E., Sanchez B.N., Cantonwine D., Lamadrid-Figueroa H., Schnaas L., Ettinger A.S., Hernandez-Avila M., Hu H., Tellez-Rojo M.M. (2011). Prenatal lead exposure and weight of 0- to 5-year-old children in Mexico city. Environ. Health Perspect..

[22] Genchi G., Sinicropi M.S., Lauria G., Carocci A., Catalano A. (2020). The Effects of Cadmium Toxicity. Int. J. Environ. Res. Public. Health.

[23] Zeng T., Liang Y., Chen J., Cao G., Yang Z., Zhao X., Tian J., Xin X., Lei B., Cai Z. (2021). Urinary metabolic characterization with nephrotoxicity for residents under cadmium exposure. Environ. Int..

[24] Freire C., Amaya E., Gil F., Murcia M., S L.L., Casas M., Vrijheid M., Lertxundi A., Irizar A., Fernandez-Tardon G. (2019). Placental metal concentrations and birth outcomes: The Environment and Childhood (INMA) project. Int. J. Hyg. Environ. Health.

[25] Geng H.X., Wang L. (2019). Cadmium: Toxic effects on placental and embryonic development. Environ. Toxicol. Pharmacol..

[26] Chandravanshi L., Shiv K., Kumar S. (2021). Developmental toxicity of cadmium in infants and children: A review. Environ. Anal. Health Toxicol..

[27] Watson C.V., Lewin M., Ragin-Wilson A., Jones R., Jarrett J.M., Wallon K., Ward C., Hilliard N., Irvin-Barnwell E. (2020). Characterization of trace elements exposure in pregnant women in the United States, NHANES 1999–2016. Environ. Res..

[28] Kippler M., Engstrom K., Mlakar S.J., Bottai M., Ahmed S., Hossain M.B., Raqib R., Vahter M., Broberg K. (2013). Sex-specific effects of early life cadmium exposure on DNA methylation and implications for birth weight. Epigenetics.

[29] Concha G., Vogler G., Lezcano D., Nermell B., Vahter M. (1998). Exposure to inorganic arsenic metabolites during early human development. Toxicol. Sci..

[30] Nyanza E.C., Dewey D., Manyama M., Martin J.W., Hatfield J., Bernier F.P. (2020). Maternal exposure to arsenic and mercury and associated risk of adverse birth outcomes in small-scale gold mining communities in Northern Tanzania. Environ. Int..

[31] Fei D.L., Koestler D.C., Li Z., Giambelli C., Sanchez-Mejias A., Gosse J.A., Marsit C.J., Karagas M.R., Robbins D.J. (2013). Association between In Utero arsenic exposure, placental gene expression, and infant birth weight: A US birth cohort study. Environ. Health.

[32] Liu H., Lu S., Zhang B., Xia W., Liu W., Peng Y., Zhang H., Wu K., Xu S., Li Y. (2018). Maternal arsenic exposure and birth outcomes: A birth cohort study in Wuhan, China. Environ. Pollut..

[33] Fry R.C., Navasumrit P., Valiathan C., Svensson J.P., Hogan B.J., Luo M., Bhattacharya S., Kandjanapa K., Soontararuks S., Nookabkaew S. (2007). Activation of inflammation/NF-kappaB signaling in infants born to arsenic-exposed mothers. PLoS Genet..

[34] Smith A.H., Steinmaus C.M. (2009). Health effects of arsenic and chromium in drinking water: Recent human findings. Annu. Rev. Public. Health.

[35] Kim B., Shah S., Park H.S., Hong Y.C., Ha M., Kim Y., Kim B.N., Kim Y., Ha E.H. (2020). Adverse effects of prenatal mercury exposure on neurodevelopment during the first 3 years of life modified by early growth velocity and prenatal maternal folate level. Environ. Res..

[36] Al-Saleh I., Al-Rouqi R., Obsum C.A., Shinwari N., Mashhour A., Billedo G., Al-Sarraj Y., Rabbah A. (2014). Mercury (Hg) and oxidative stress status in healthy mothers and its effect on birth anthropometric measures. Int. J. Hyg. Environ. Health.

[37] Xue F., Holzman C., Rahbar M.H., Trosko K., Fischer L. (2007). Maternal fish consumption, mercury levels, and risk of preterm delivery. Environ. Health Perspect..

[38] Sattler B., Randall K.S., Choiniere D. (2012). Reducing hazardous chemical exposures in the neonatal intensive care unit: A new role for nurses. Crit. Care Nurs. Q..

[39] Boucher O., Muckle G., Jacobson J.L., Carter R.C., Kaplan-Estrin M., Ayotte P., Dewailly E., Jacobson S.W. (2014). Domain-specific effects of prenatal exposure to PCBs, mercury, and lead on infant cognition: Results from the Environmental Contaminants and Child Development Study in Nunavik. Environ. Health Perspect..

[40] Chen Z., Myers R., Wei T., Bind E., Kassim P., Wang G., Ji Y., Hong X., Caruso D., Bartell T. (2014). Placental transfer and concentrations of cadmium, mercury, lead, and selenium in mothers, newborns, and young children. J. Expo. Sci. Environ. Epidemiol..

[41] Chuang H.Y., Schwartz J., Gonzales-Cossio T., Lugo M.C., Palazuelos E., Aro A., Hu H., Hernandez-Avila M. (2001). Interrelations of lead levels in bone, venous blood, and umbilical cord blood with exogenous lead exposure through maternal plasma lead in peripartum women. Environ. Health Perspect..

[42] Jelliffe-Pawlowski L.L., Miles S.Q., Courtney J.G., Materna B., Charlton V. (2006). Effect of magnitude and timing of maternal pregnancy blood lead (Pb) levels on birth outcomes. J. Perinatol..

[43] Perkins M., Wright R.O., Amarasiriwardena C.J., Jayawardene I., Rifas-Shiman S.L., Oken E. (2014). Very low maternal lead level in pregnancy and birth outcomes in an eastern Massachusetts population. Ann. Epidemiol..

[44] Liu J., Chen Y., Gao D., Jing J., Hu Q. (2014). Prenatal and postnatal lead exposure and cognitive development of infants followed over the first three years of life: A prospective birth study in the Pearl River Delta region, China. Neurotoxicology.

[45] Virgolini M.B., Rossi-George A., Weston D., Cory-Slechta D.A. (2008). Influence of low level maternal Pb exposure and prenatal stress on offspring stress challenge responsivity. Neurotoxicology.

[46] Jacob B., Ritz B., Heinrich J., Hoelscher B., Wichmann H.E. (2000). The effect of low-level blood lead on hematologic parameters in children. Environ. Res..

[47] Serrani R.E., Gioia I.A., Corchs J.L. (1997). Lead effects on structural and functional cellular parameters in human red cells from a prenatal hematopoiesis stage. Biometals.

[48] Pirkle J.L., Brody D.J., Gunter E.W., Kramer R.A., Paschal D.C., Flegal K.M., Matte T.D. (1994). The decline in blood lead levels in the United States. The National Health and Nutrition Examination Surveys (NHANES). JAMA.

[49] Berkowitz Z., Price-Green P., Bove F.J., Kaye W.E. (2006). Lead exposure and birth outcomes in five communities in Shoshone County, Idaho. Int. J. Hyg. Environ. Health.

[50] Sowers M., Jannausch M., Scholl T., Li W., Kemp F.W., Bogden J.D. (2002). Blood lead concentrations and pregnancy outcomes. Arch. Environ. Health.

[51] Satin K.P., Neutra R.R., Guirguis G., Flessel P. (1991). Umbilical cord blood lead levels in California. Arch. Environ. Health.

[52] Dettwiler M., Flynn A.C., Rigutto-Farebrother J. (2023). Effects of Non-Essential “Toxic” Trace Elements on Pregnancy Outcomes: A Narrative Overview of Recent Literature Syntheses. Int. J. Environ. Res. Public. Health.

[53] Mertz W. (1981). The essential trace elements. Science.

[54] Fraga C.G. (2005). Relevance, essentiality and toxicity of trace elements in human health. Mol. Aspects Med..

[55] Jariwala M., Suvarna S., Kumar G.K., Amin A., Udas A.C. (2014). Study of the Concentration of Trace Elements Fe, Zn, Cu, Se and Their Correlation in Maternal Serum, Cord Serum and Colostrums. Indian. J. Clin. Bioche.

[56] Claus Henn B., Ettinger A.S., Schwartz J., Tellez-Rojo M.M., Lamadrid-Figueroa H., Hernandez-Avila M., Schnaas L., Amarasiriwardena C., Bellinger D.C., Hu H. (2010). Early postnatal blood manganese levels and children’s neurodevelopment. Epidemiology.

[57] Boskabadi H., Maamouri G., Rezagholizade Omran F., Mafinejad S., Tara F., Rayman M.P., Ghayour-Mobarhan M., Sahebkar A., Tavallaie S., Shakeri M.T. (2012). Effect of prenatal selenium supplementation on cord blood selenium and lipid profile. Pediatr. Neonatol..

[58] Rahbar M.H., Samms-Vaughan M., Dickerson A.S., Hessabi M., Bressler J., Desai C.C., Shakespeare-Pellington S., Reece J.A., Morgan R., Loveland K.A. (2015). Concentration of lead, mercury, cadmium, aluminum, arsenic and manganese in umbilical cord blood of Jamaican newborns. Int. J. Environ. Res. Public. Health.

[59] Dai Y., Zhang J., Qi X., Wang Z., Zheng M., Liu P., Jiang S., Guo J., Wu C., Zhou Z. (2021). Cord Blood Manganese Concentrations in Relation to Birth Outcomes and Childhood Physical Growth: A Prospective Birth Cohort Study. Nutrients.

[60] Yamamoto M., Eguchi A., Sakurai K., Nakayama S.F., Sekiyama M., Mori C., Kamijima M., Japan Environment C.s.S.G. (2022). Longitudinal analyses of maternal and cord blood manganese levels and neurodevelopment in children up to 3 years of age: The Japan Environment and Children’s Study (JECS). Environ. Int..

[61] Lorenzo Alonso M.J., Bermejo Barrera A., Cocho de Juan J.A., Fraga Bermudez J.M., Bermejo Barrera P. (2005). Selenium levels in related biological samples: Human placenta, maternal and umbilical cord blood, hair and nails. J. Trace Elem. Med. Biol..

[62] Gilman C.L., Soon R., Sauvage L., Ralston N.V., Berry M.J. (2015). Umbilical cord blood and placental mercury, selenium and selenoprotein expression in relation to maternal fish consumption. J. Trace Elem. Med. Biol..

[63] Archimbaud Y., Grillon G., Poncy J.L., Masse R. (1992). Se-75 Transfer Via Placenta and Milk, Distribution and Retention in Fetal, Young and Adult-Rat. Radiat. Prot. Dosim..

[64] Jandial V., Henderson P., Macgillivray I. (1976). Placental-Transfer of Radioactive Selenomethionine in Late Pregnancy. Eur. J. Obstet. Gyn R. B.

[65] Cerna M., Spevackova V., Cejchanova M., Benes B., Rossner P., Bavorova H., Ocadlikova D., Smid J., Kubinova R. (1997). Population-based biomonitoring in the Czech Republic--the system and selected results. Sci. Total Environ..

[66] Robkin M.A., Swanson D.R., Shepard T.H. (1973). Trace Metal Concentrations in Human Fetal Livers. T Am. Nucl. Soc..

[67] Kim H., Harrison F.E., Aschner M., Bowman A.B. (2022). Exposing the role of metals in neurological disorders: A focus on manganese. Trends Mol. Med..

[68] Amoros R., Murcia M., Gonzalez L., Soler-Blasco R., Rebagliato M., Iniguez C., Carrasco P., Vioque J., Broberg K., Levi M. (2019). Maternal copper status and neuropsychological development in infants and preschool children. Int. J. Hyg. Environ. Health.

[69] Weyde K.V.F., Winterton A., Suren P., Andersen G.L., Vik T., Biele G., Knutsen H.K., Thomsen C., Meltzer H.M., Skogheim T.S. (2023). Association between gestational levels of toxic metals and essential elements and cerebral palsy in children. Front. Neurol..

[70] Kumar V., Kalita J., Misra U.K., Bora H.K. (2015). A study of dose response and organ susceptibility of copper toxicity in a rat model. J. Trace Elem. Med. Biol..

[71] Kumar V., Kalita J., Bora H.K., Misra U.K. (2016). Relationship of antioxidant and oxidative stress markers in different organs following copper toxicity in a rat model. Toxicol. Appl. Pharmacol..

[72] Chen J., Gao X., Zheng C., Zhang C., Li P., He K., Liu G., Huang X., Liu J., Xie Y. (2023). Low-dose Cu exposure enhanced alpha-synuclein accumulation associates with mitochondrial impairments in mice model of Parkinson’s disease. Toxicol. Lett..

[73] Monangi N.K., Xu H., Fan Y.M., Khanam R., Khan W., Deb S., Pervin J., Price J.T., Kaur L., INTERBIO-21st Study Consortium (2024). Association of maternal prenatal copper concentration with gestational duration and preterm birth: A multicountry meta-analysis. Am. J. Clin. Nutr..

[74] Cengiz B., Soylemez F., Ozturk E., Cavdar A.O. (2004). Serum zinc, selenium, copper, and lead levels in women with second-trimester induced abortion resulting from neural tube defects: A preliminary study. Biol. Trace Elem. Res..

[75] Leotsinidis M., Alexopoulos A., Kostopoulou-Farri E. (2005). Toxic and essential trace elements in human milk from Greek lactating women: Association with dietary habits and other factors. Chemosphere.

[76] Davidson P.W., Palumbo D., Myers G.J., Cox C., Shamlaye C.F., Sloane-Reeves J., Cernichiari E., Wilding G.E., Clarkson T.W. (2000). Neurodevelopmental outcomes of Seychellois children from the pilot cohort at 108 months following prenatal exposure to methylmercury from a maternal fish diet. Environ. Res..

[77] Dorea J.G., Donangelo C.M. (2006). Early (in uterus and infant) exposure to mercury and lead. Clin. Nutr..

[78] Dursun A., Yurdakok K., Yalcin S.S., Tekinalp G., Aykut O., Orhan G., Morgil G.K. (2016). Maternal risk factors associated with lead, mercury and cadmium levels in umbilical cord blood, breast milk and newborn hair. J. Matern. Fetal Neonatal Med..

[79] Yalçin S.S., Yurdakök K., Yalçin S., Engür-Karasimav D., Coşkun T. (2010). Maternal and environmental determinants of breast-milk mercury concentrations. Turk. J. Pediatr..

[80] Yang W., Han N., Jiao M., Chang X., Liu J., Zhou Q., Wang H.J. (2023). Maternal diet quality during pregnancy and its influence on low birth weight and small for gestational age: A birth cohort in Beijing, China. Br. J. Nutr..

[81] Stone J., Sutrave P., Gascoigne E., Givens M.B., Fry R.C., Manuck T.A. (2021). Exposure to toxic metals and per- and polyfluoroalkyl substances and the risk of preeclampsia and preterm birth in the United States: A review. Am. J. Obstet. Gynecol. MFM.

[82] Yang J., Huo W., Zhang B., Zheng T., Li Y., Pan X., Liu W., Chang H., Jiang M., Zhou A. (2016). Maternal urinary cadmium concentrations in relation to preterm birth in the Healthy Baby Cohort Study in China. Environ. Int..

[83] Takci S., Asci A., Erkekoglu P., Yiğit S., Kocer-Gumusel B., Yurdakök M. (2019). Lead and Mercury Levels in Preterm Infants Before and After Blood Transfusions. Biol. Trace Elem. Res..

[84] Bearer C.F., O’Riordan M.A., Powers R. (2000). Lead exposure from blood transfusion to premature infants. J. Pediatr..

[85] Salazar-Martinez E., Romano-Riquer P., Yanez-Marquez E., Longnecker M.P., Hernandez-Avila M. (2004). Anogenital distance in human male and female newborns: A descriptive, cross-sectional study. Environ. Health.

[86] Akin Y., Ercan O., Telatar B., Tarhan F. (2011). Penile size in term newborn infants. Turk. J. Pediatr..

[87] Lozano M., Murcia M., Soler-Blasco R., Casas M., Zubero B., Riutort-Mayol G., Gil F., Olmedo P., Grimalt J.O., Amoros R. (2022). Exposure to metals and metalloids among pregnant women from Spain: Levels and associated factors. Chemosphere.

[88] Song J., Wang X., Huang Q., Wei C., Yang D., Wang C., Fan K., Cheng S., Guo X., Wang J. (2024). Predictors of urinary heavy metal concentrations among pregnant women in Jinan, China. J. Trace Elem. Med. Biol..

[89] Rudge C.V., Rollin H.B., Nogueira C.M., Thomassen Y., Rudge M.C., Odland J.O. (2009). The placenta as a barrier for toxic and essential elements in paired maternal and cord blood samples of South African delivering women. J. Environ. Monit..

[90] Al-Saleh I., Al-Rouqi R., Alnuwaysir H., Aldhalaan H., Alismail E., Binmanee A., Hawari A., Alhazzani F., Bin Jabr M. (2023). Exposure of preterm neonates to toxic metals during their stay in the Neonatal Intensive Care Unit and its impact on neurodevelopment at 2 months of age. J. Trace Elem. Med. Biol..

[91] Iwai-Shimada M., Kameo S., Nakai K., Yaginuma-Sakurai K., Tatsuta N., Kurokawa N., Nakayama S.F., Satoh H. (2019). Exposure profile of mercury, lead, cadmium, arsenic, antimony, copper, selenium and zinc in maternal blood, cord blood and placenta: The Tohoku Study of Child Development in Japan. Environ. Health Prev. Med..

[92] Kopp R.S., Kumbartski M., Harth V., Bruning T., Kafferlein H.U. (2012). Partition of metals in the maternal/fetal unit and lead-associated decreases of fetal iron and manganese: An observational biomonitoring approach. Arch. Toxicol..

[93] Arbuckle T.E., Liang C.L., Morisset A.S., Fisher M., Weiler H., Cirtiu C.M., Legrand M., Davis K., Ettinger A.S., Fraser W.D. (2016). Maternal and fetal exposure to cadmium, lead, manganese and mercury: The MIREC study. Chemosphere.

[94] Garí M., Grzesiak M., Krekora M., Kaczmarek P., Jankowska A., Król A., Kaleta D., Jerzyńska J., Janasik B., Kuraś R. (2022). Prenatal exposure to neurotoxic metals and micronutrients and neurodevelopmental outcomes in early school age children from Poland. Environ. Res..

[95] Dahiri B., Martín-Carrasco I., Carbonero-Aguilar P., Cerrillos L., Ostos R., Fernández-Palacín A., Bautista J., Moreno I. (2023). Monitoring of metals and metalloids from maternal and cord blood samples in a population from Seville (Spain). Sci. Total Environ..

[96] Zhang T., Wang X., Luo Z.C., Liu J., Chen Y., Fan P., Ma R., Ma J., Luo K., Yan C.H. (2023). Maternal blood concentrations of toxic metal(loid)s and trace elements from preconception to pregnancy and transplacental passage to fetuses. Ecotoxicol. Environ. Saf..

[97] Garcia-Esquinas E., Perez-Gomez B., Fernandez-Navarro P., Fernandez M.A., de Paz C., Perez-Meixeira A.M., Gil E., Iriso A., Sanz J.C., Astray J. (2013). Lead, mercury and cadmium in umbilical cord blood and its association with parental epidemiological variables and birth factors. BMC Public. Health.

[98] Grzesik-Gasior J., Sawicki J., Pieczykolan A., Bien A. (2023). Content of selected heavy metals in the umbilical cord blood and anthropometric data of mothers and newborns in Poland: Preliminary data. Sci. Rep..

[99] Bocca B., Ruggieri F., Pino A., Rovira J., Calamandrei G., Martinez M.A., Domingo J.L., Alimonti A., Schuhmacher M. (2019). Human biomonitoring to evaluate exposure to toxic and essential trace elements during pregnancy. Part A. concentrations in maternal blood, urine and cord blood. Environ. Res..

[100] Guo X., Song J., Wang X., Huang Q., Wei C., Yang Y., Li N., Cheng S., Li J., Li Q. (2024). Urinary concentrations of mineral elements and their predictors in pregnant women in Jinan, China. J. Trace Elem. Med. Biol..

[101] Gundacker C., Frohlich S., Graf-Rohrmeister K., Eibenberger B., Jessenig V., Gicic D., Prinz S., Wittmann K.J., Zeisler H., Vallant B. (2010). Perinatal lead and mercury exposure in Austria. Sci. Total Environ..

[102] Lagerkvist B.I., Sandberg S., Frech W., Jin T., Nordberg G.F. (1996). Is placenta a good indicator of cadmium and lead exposure?. Arch. Environ. Health.

[103] Gundacker C., Hengstschlager M. (2012). The role of the placenta in fetal exposure to heavy metals. Wien. Med. Wochenschr..

[104] Nazemi L., Shariat M., Chamari M., Chahardoli R., Asgarzadeh L., Seighali F. (2015). Comparison of Maternal and Umbilical Cord Blood Selenium Levels in Low and Normal Birth Weight Neonates. J. Family Reprod. Health.

[105] Sanchez C., Lopez-Jurado M., Aranda P., Llopis J. (2010). Plasma levels of copper, manganese and selenium in an adult population in southern Spain: Influence of age, obesity and lifestyle factors. Sci. Total Environ..

[106] Gaspari L., Tessier B., Paris F., Bergougnoux A., Hamamah S., Sultan C., Kalfa N. (2021). Endocrine-Disrupting Chemicals and Disorders of Penile Development in Humans. Sex. Dev..

[107] Gaspari L., Sampaio D.R., Paris F., Audran F., Orsini M., Neto J.B., Sultan C. (2012). High prevalence of micropenis in 2710 male newborns from an intensive-use pesticide area of Northeastern Brazil. Int. J. Androl..

[108] Nelson C.P., Park J.M., Wan J., Bloom D.A., Dunn R.L., Wei J.T. (2005). The increasing incidence of congenital penile anomalies in the United States. J. Urol..

[109] Martin M.B., Reiter R., Johnson M., Shah M.S., Iann M.C., Singh B., Richards J.K., Wang A., Stoica A. (2007). Effects of tobacco smoke condensate on estrogen receptor-alpha gene expression and activity. Endocrinology.

[110] dos Santos N.R., Rodrigues J.L.G., Bandeira M.J., Anjos A.L.d.S., Araújo C.d.F.S., Adan L.F.F., Menezes-Filho J.A. (2019). Manganese exposure and association with hormone imbalance in children living near a ferro-manganese alloy plant. Environ. Res..

[111] Dos Santos N.R., Rodrigues J.L.G., Bandeira M.J., Anjos A., Araújo C., Adan L.F.F., Menezes-Filho J.A. (2022). Manganese and Lead Exposure and Early Puberty Onset in Children Living near a Ferromanganese Alloy Plant. Int. J. Environ. Res. Public. Health.

[112] Gomes-Silva A.P., Medeiros P.D.C., Silva L.N.D., Santiago M., Trevizani T.H., Figueira R.C.L., Moreira L.B., Perobelli J.E. (2025). Exposure to manganese during late intrauterine development and lactation: Long-term effects on male reproductive function. Environ. Toxicol. Pharmacol..

[113] Zheng G., Zhong H., Guo Z., Wu Z., Zhang H., Wang C., Zhou Y., Zuo Z. (2014). Levels of heavy metals and trace elements in umbilical cord blood and the risk of adverse pregnancy outcomes: A population-based study. Biol. Trace Elem. Res..

[114] Zinia S.S., Yang K.H., Lee E.J., Lim M.N., Kim J., Kim W.J. (2023). Effects of heavy metal exposure during pregnancy on birth outcomes. Sci. Rep..

[115] Rahman A., Al-Rashidi H.A., Khan A.R. (2012). Association of maternal blood lead level during pregnancy with child blood lead level and pregnancy outcome in Kuwait. Ecol. Food Nutr..

[116] Rahman A., Kumarathasan P., Gomes J. (2016). Infant and mother related outcomes from exposure to metals with endocrine disrupting properties during pregnancy. Sci. Total Environ..

[117] Xie X., Ding G., Cui C., Chen L., Gao Y., Zhou Y., Shi R., Tian Y. (2013). The effects of low-level prenatal lead exposure on birth outcomes. Environ. Pollut..

[118] Marini M., Angouria-Tsorochidou E., Caro D., Thomsen M. (2021). Daily intake of heavy metals and minerals in food–A case study of four Danish dietary profiles. J. Clean. Prod..

[119] Muntau A.C., Streiter M., Kappler M., Röschinger W., Schmid I., Rehnert A., Schramel P., Roscher A.A. (2002). Age-related reference values for serum selenium concentrations in infants and children. Clin. Chem..

[120] Bebars G., Kamel B., Allam E. (2018). Comparison between preterm and full term neonatal cord selenium in correlation to maternal serum selenium levels. Egypt. Pediatr. Assoc. Gaz..

[121] Monangi N., Xu H., Khanam R., Khan W., Deb S., Pervin J., Price J.T., Kennedy S.H., Al Mahmud A., Fan Y. (2021). Association of maternal prenatal selenium concentration and preterm birth: A multicountry meta-analysis. BMJ Glob. Health.

[122] Lewandowska M., Sajdak S., Lubiński J. (2019). The Role of Early Pregnancy Maternal Selenium Levels on the Risk for Small-for-Gestational Age Newborns. Nutrients.

[123] Lyons G.H., Judson G.J., Stangoulis J.C., Palmer L.T., Jones J.A., Graham R.D. (2004). Trends in selenium status of South Australians. Med. J. Aust..

[124] Liu X., Zhang Y., Piao J., Mao D., Li Y., Li W., Yang L., Yang X. (2017). Reference Values of 14 Serum Trace Elements for Pregnant Chinese Women: A Cross-Sectional Study in the China Nutrition and Health Survey 2010–2012. Nutrients.

