# Supplementary material for: Perinatal Exposure to Heavy Metals and Trace Elements of Preterm Neonates in the NICU: A Toxicological Study Using Multiple Biomatrices
Source: Toxics. 2025 Oct 20;13(10):898. doi: 10.3390/toxics13100898 (PMC12568181; doi:10.3390/toxics13100898)
Supplement: Supplementary file 1 [file toxics-13-00898-s001.zip › toxics-3909909-supplementary.pdf]

Supplementary Tables

Table S1. Correlations of Maternal Urinary Heavy Metal and Trace Element Levels with Mother-Infant Characteristics

		Maternal age	*Parity*	*Gestational week*	*Maternal BMI*	*Pregnancy Weight gain (123)*	*Pregnancy Weight gain*	*BW perc.*	*HC perc.*	*AGD*	*SPL*	MuPb	MuAs	MuCd	MuHg	MuMn	MuSe
MuPb	r_s	-0.18	-0.12	-0.05	0.04	-0.08	0.02	-0.06	0.05	0.18	0.15						
	p	0.280	0.474	0.771	0.810	0.615	0.918	0.732	0.771	0.277	0.478						
MuAs	r_s	0.06	0.09	0.06	0.22	0.13	**-0.37**	0.23	0.11	0.28	0.20	-0.12					
	p	0.696	0.564	0.725	0.184	0.432	**0.020**	0.146	0.498	0.075	0.343	0.471					
MuCd	r_s	0.19	**0.32**	-0.09	-0.14	0.13	-0.10	0.05	-0.08	0.15	0.18	-0.16	**0.31**				
	p	0.240	**0.044**	0.575	0.379	0.427	0.554	0.782	0.617	0.348	0.396	0.331	**0.048**				
MuHg	r_s	-0.24	**-0.36**	0.08	-0.24	-0.04	0.16	-0.11	0.00	0.23	-0.13	0.07	0.16	0.06			
	p	0.139	**0.022**	0.614	0.135	0.823	0.314	0.503	0.978	0.156	0.547	0.683	0.333	0.694			
MuMn	r_s	-0.27	-0.20	-0.22	-0.31	0.07	0.23	-0.05	0.11	-0.09	**-0.46**	0.17	-0.15	-0.21	**0.40**		
	p	0.089	0.224	0.166	0.054	0.662	0.150	0.779	0.514	0.571	**0.020**	0.309	0.346	0.198	**0.011**		
MuSe	r_s	**0.41**	0.20	-0.18	-0.04	-0.09	-0.08	0.01	-0.04	0.16	0.16	0.28	0.12	0.20	-0.09	-0.13	
	p	**0.009**	0.214	0.258	0.829	0.592	0.605	0.937	0.823	0.327	0.460	0.082	0.481	0.217	0.578	0.438	
MuCu	r_s	0.08	0.11	-0.14	-0.03	0.06	-0.10	-0.11	-0.18	0.22	0.28	0.28	**0.37**	0.30	-0.09	-0.10	**0.33**
	p	0.607	0.501	0.382	0.875	0.731	0.558	0.517	0.270	0.167	0.184	0.086	**0.018**	0.060	0.563	0.550	**0.037**

p: significance (<0.05), r_s: Spearman correlation coefficient

Mu: Maternal urine, Pb: Lead, As: Arsenic, Cd: Cadmium, Hg: Mercury, Mn: Manganese, Se: Selenium, Cu: Copper, BW perc: Birthweight percentile, HC perc: Head circumference percentile, AGD: Anogenital distance, SPL: Stretched penile length

Table S2. Correlations of Cord Blood Heavy Metal and Trace Element Levels with Mother-Infant Characteristics

		Maternal age	*Parity*	*Gestational week*	*Maternal BMI*	*Pregnancy Weight gain (123)*	*Pregnancy Weight gain*	*BW perc.*	*HC perc.*	*AGD*	*SPL*	Cord Pb	Cord As	Cord Cd	Cord Hg	Cord Mn	Cord Se
Cord_Pb	r_s	-0.10	-0.02	0.00	0.18	-0.03	0.12	-0.04	0.21	-0.11	-0.08						
	p	0.546	0.914	1.000	0.278	0.854	0.476	0.831	0.198	0.505	0.704						
Cord_As	r_s	0.21	0.03	-0.14	-0.27	-0.12	0.13	-0.11	0.04	-0.02	-0.12	0.06					
	p	0.186	0.841	0.391	0.094	0.471	0.412	0.509	0.827	0.927	0.581	0.697					
Cord_Cd	r_s	-0.14	-0.02	-0.12	**0.39**	0.17	0.05	0.09	0.19	-0.13	-0.09	0.14	0.21				
	p	0.374	0.897	0.449	**0.012**	0.302	0.753	0.591	0.252	0.428	0.686	0.380	0.189				
Cord_Hg	r_s	-0.02	-0.03	0.08	-0.02	-0.31	-0.22	0.11	-0.03	-0.24	-0.07	0.27	-0.06	-0.16			
	p	0.888	0.840	0.642	0.891	0.051	0.175	0.497	0.867	0.129	0.743	0.087	0.731	0.336			
Cord_Mn	r_s	0.05	0.28	-0.12	-0.20	-0.09	-0.12	-0.08	0.04	-0.15	**-0.40**	0.13	0.12	-0.02	0.14		
	p	0.777	0.082	0.453	0.229	0.590	0.479	0.624	0.822	0.369	**0.048**	0.413	0.459	0.881	0.400		
Cord_Se	r_s	-0.19	-0.19	**0.42**	0.02	0.10	0.23	-0.19	-0.08	-0.07	0.04	0.07	0.01	-0.07	-0.01	0.29	
	p	0.240	0.239	**0.008**	0.929	0.535	0.160	0.252	0.621	0.675	0.848	0.681	0.954	0.660	0.978	0.071	
Cord_Cu	r_s	0.13	0.26	0.01	0.11	0.23	0.17	-0.03	-0.07	-0.11	0.13	-0.12	0.26	**0.39**	-0.13	0.00	0.02
	p	0.427	0.111	0.931	0.487	0.147	0.285	0.873	0.675	0.483	0.553	0.465	0.102	**0.014**	0.417	0.989	0.925

p: significance (<0.05), r_s: Spearman correlation coefficient

Mu: Maternal urine, Pb: Lead, As: Arsenic, Cd: Cadmium, Hg: Mercury, Mn: Manganese, Se: Selenium, Cu: Copper, BW perc: Birthweight percentile, HC perc: Head circumference percentile, AGD: Anogenital distance, SPL: Stretched penile length

Table S3.Correlations of First Neonatal Urinary Heavy Metal and Trace Element Levels with Mother-Infant Characteristics

		Maternal age	Number of births	Gestational week	AGD	SPL	BW perc	HC perc	NuPb	NuAs	NuCd	NuHg	NuMn	NuSe
NuPb1	r_s	0.21	0.26	-0.12	0.00	-0.02	**0.36**	0.24						
	p	0.194	0.103	0.476	0.981	0.939	**0.022**	0.131						
NuAs1	r_s	-0.03	-0.12	-0.04	-0.10	-0.35	0.00	-0.01	-0.21					
	p	0.875	0.455	0.825	0.542	0.084	0.991	0.930	0.195					
NuCd1	r_s	-0.09	-0.12	-0.11	0.26	-0.09	0.19	0.16	-0.01	0.16				
	p	0.578	0.474	0.483	0.110	0.678	0.247	0.328	0.967	0.312				
NuHg1	r_s	-0.02	0.00	0.04	**-0.33**	**-0.48**	-0.13	-0.20	0.12	0.06	-0.13			
	p	0.928	0.993	0.832	**0.040**	**0.016**	0.414	0.223	0.457	0.697	0.431			
NuMn1	r_s	-0.03	0.19	-0.22	0.07	0.04	0.31	0.22	0.18	-0.03	**0.36**	-0.07		
	p	0.877	0.243	0.178	0.675	0.843	0.056	0.165	0.281	0.880	**0.022**	0.657		
NuSe1	r_s	-0.27	-0.11	**0.35**	-0.28	-0.10	-0.08	-0.12	-0.03	0.20	-0.15	-0.01	-0.04	
	p	0.090	0.510	**0.027**	0.083	0.652	0.634	0.474	0.872	0.227	0.360	0.967	0.789	
NuCu1	r_s	0.01	0.01	-0.13	0.19	-0.05	0.12	0.09	0.16	-0.14	0.12	0.04	0.20	0.18
	p	0.931	0.967	0.416	0.237	0.831	0.453	0.565	0.325	0.401	0.453	0.820	0.221	0.258

p: significance (<0.05), r_s: Spearman correlation coefficient

Nu: Neonatal urine, Pb: Lead, As: Arsenic, Cd: Cadmium, Hg: Mercury, Mn: Manganese, Se: Selenium, Cu: Copper, BW perc: Birthweight percentile, HC perc: Head circumference percentile, AGD: Anogenital distance,, SPL: Stretched penile length

Table S4.Correlations of Second Neonatal Urinary Heavy Metal and Trace Element Levels with Mother-Infant Characteristics

		Maternal age	Number of births	Gestational week	AGD	SPL	BW perc	HC per	NuPb2	NuAs2	NuCd2	NuHg2	NuMn2	NuSe2
NuPb2	r_s	**0.33**	0.30	-0.12	0.01	-0.06	**0.38**	0.31						
	p	**0.037**	0.064	0.462	0.967	0.769	**0.016**	0.051						
NuAs2	r_s	-0.06	-0.25	0.12	0.02	-0.18	-0.18	-0.12	-0.13					
	p	0.717	0.126	0.473	0.899	0.401	0.277	0.456	0.443	.				
NuCd2	r_s	0.04	0.10	-0.16	0.03	-0.04	0.19	0.14	0.11	-0.24				
	p	0.815	0.541	0.324	0.835	0.863	0.235	0.404	0.509	0.129	.			
NuHg2	r_s	-0.03	0.05	-0.01	-0.26	**-0.58**	-0.09	-0.18	0.05	0.07	-0.04			
	p	0.85	0.766	0.958	0.11	**0.003**	0.570	0.256	0.780	0.669	0.820	.		
NuMn2	r_s	0.02	0.18	**-0.37**	-0.03	-0.21	0.21	0.22	0.40	-0.19	0.07	-0.04		
	p	0.906	0.271	**0.018**	0.873	0.307	0.188	0.167	0.011	0.236	0.666	0.819	.	
NuSe2	r_s	-0.19	-0.17	0.28	-0.19	-0.36	-0.02	-0.10	0.22	-0.06	0.14	0.22	0.03	
	p	0.231	0.294	0.080	0.252	0.076	0.888	0.552	0.175	0.709	0.397	0.173	0.841	.
NuCu2	r_s	0.22	0.18	-0.26	0.13	0.06	-0.18	0.08	0.19	-0.04	0.10	0.00	0.02	0.00
	p	0.17	0.263	0.102	0.417	0.771	0.257	0.639	0.236	0.796	0.526	0.996	0.885	0.989

p: significance (<0.05), rs: Spearman correlation coefficient

Nu: Neonatal urine, Pb: Lead, As: Arsenic, Cd: Cadmium, Hg: Mercury, Mn: Manganese, Se: Selenium, Cu: Copper, BW perc: Birthweight percentile, HC perc: Head circumference percentile, AGD: Anogenital distance,, SPL: Stretched penile length
